# Recent progress in targeting cancer

**DOI:** 10.18632/aging.100421

**Published:** 2011-12-31

**Authors:** Zoya N. Demidenko, James A. McCubrey

**Affiliations:** ^1^ Department of Cell Stress Biology, Roswell Park Cancer Institute, Elm and Carlton Streets, Buffalo, NY, 14263, USA; ^2^ Department of Microbiology and Immunology, Brody School of Medicine, East Carolina University, Greenville, NC 27834, USA

**Keywords:** cancer, target, therapy, leukemia, anticancer drugs

## Abstract

In recent years, numerous new targets have been identified and new experimental therapeutics have been developed. Importantly, existing non-cancer drugs found novel use in cancer therapy. And even more importantly, new original therapeutic strategies to increase potency, selectivity and decrease detrimental side effects have been evaluated. Here we review some recent advances in targeting cancer.

In 1977, Andrzej “Andrew” V. Schally won Nobel Prize in medicine for his research into peptide hormone production in the brain. He described the neurohormone GnRH and other releasing hormones (RH). As initially unexpected application, agonists and antagonists of these hormones have become investigational anti-cancer agents [1-3]. As further developments, Schally and coworkers described targeting gastrin releasing peptide receptors. Gastrin-releasing peptide (GRP) is involved in cancer growth and GRP receptors are expressed in a variety of cancer cells and have limited distribution in normal human tissue. Thus inhibition of GRP receptors represents an attractive target for pharmacological treatment of certain human malignancies [4]. Also, MZ-5-156, an antagonist of growth hormone-releasing hormone (GHRH), decreased cell proliferation and activated AMPK and inhibited Akt, the mammalian target of rapamycin (mTOR) and its downstream target eIF4E which controls protein synthesis and cell growth [5]. GHRH antagonists also caused cell cycle arrest and apoptosis in human colon cancer cells [6, 7].

Yet, this is only one of hundreds examples for new therapeutic targets and new types of drugs that have been developed recently in cellular and animal models. Searche for new targets has continued with many promising lead compounds identified [8-38].

Among promising targets are cancer stem cells [39-42], microRNAs [43-50], the MEK/ERK pathway [51-64] and especially its upstream activator BRAF [61, 65-67] and the NF-kB pathway [68], Myc and HIF-1 [69-72], The CtBP transcriptional corepressors [73], Polycomb group (PcG) proteins [74], autophagy [75-77], translation [78], the proteasome [35], HSP70 [79, 80], Hsp90 [81-84], the AMPK-FoxO3A axis [85], STAT3 and MEK/ERK/BCL-2 signaling [86], the Hh signal transducer Smoothened [87], ErbBs receptor tyrosine kinases [88], and anti-apoptotic members of the Bcl-2 family, Bcl-2, Bcl-X(L) and Mcl-1 [89]. Stromal and endothelial cells are also targets [90, 91]. There are also new targets for anti-angiogenic therapy [71, 75, 78, 92-94]. Also, epithelial mesenchymal transition (EMT) is a critical mechanism for the acquisition of malignant phenotypes by epithelial cells [95]. In colorectal cancer, such cells are histologically represented by tumor buds defined as single cells or small clusters of de-differentiated tumor cells at the invasive front. These buds are also considered as targets for novel cancer therapy [96, 97]. Recently, leukocytes in the ovarian cancer microenvironment such as regulatory T cells and immature pro-angiogenic myeloid cells have been demonstrated to play a fundamental role in tumor progression and have been suggested as potential target [98]. Cdk4/6 is an attractive target for cancer therapy. Thus, a 2-aminothiazole-derived Cdk4/6 selective inhibitor, named Compound A potently inhibits Cdk4 and Cdk6 with high selectivity [99]. Among 82 human cell line examined, leukemia and lymphoma cell lines tended to be more sensitive to Compound A. In a nude rat xenograft model, Compound A inhibited cell proliferation in xenograft tumors at a plasma concentration of 510 nM. Compound A only moderately inhibited cell cycle progression of normal crypt cells in small intestine even at 5 times higher plasma concentration and did not cause immunosuppression even at 17 times higher concentration [99].

Targeting the androgen receptor also has also shown significant progress [100-103]. An interesting example is targeting androgen receptor in estrogen receptor-negative breast cancer [104]. Also, a small-molecule inhibitor of the amino-terminus domain of the androgen receptor causes regression of castrate-recurrent prostate cancer [105, 106]. Recent discoveries revealed a transcription-independent function of androgen receptor that is essential for prostate cancer cell viability and, therefore, is an ideal target for anticancer treatment. Several of the identified AR inhibitors demonstrated in vivo efficacy in mouse models of PCa and are candidates for pharmacologic optimization [107].

Among numerous new experimental therapeutics, a small-molecule inducer of polyploidy, R1530, interferes with tubulin polymerization, leads to abortive mitosis, endoreduplication and polyploidy. In the presence of R1530, polyploid cancer cells underwent apoptosis or became senescent which translated into potent in vitro and in vivo efficacy. Normal proliferating cells were resistant to R1530-induced polyploidy thus supporting the rationale for cancer therapy by induced polyploidy. BubR1 plays a key role in polyploidy induction by R1530 and could be exploited as a target for designing more specific polyploidy inducers [108]. Vosaroxin (formerly voreloxin) is a first-in-class anticancer quinolone derivative that intercalates DNA and inhibits topoisomerase II, inducing site-selective double-strand breaks (DSB), G2 arrest and apoptosis. Homologous recombination repair (HRR) is critical for recovery from DNA damage induced by both agents, identifying the potential to clinically exploit synthetic lethality [109]. Depletion of POLQ (DNA polymerase theta) renders tumor cells more sensitive to radiotherapy without effecting normal tissues, providing ways to increase therapeutic window [110]. Mycoplasma is also a target for cancer prevention [111] and therapy [112]. Among additional targets is activating transcription factor 5 (ATF5), an anti-apoptotic protein that is highly expressed in malignant glioma but not normal brain tissues, and is essential for glioma cell survival [113].

There were several developments in targeting p53 and its cousins, p73 and p63 [114-121]. Nutlin-3a is a non-genotoxic inducer of p53 and causes the transcription-independent mitochondrial p53 program of nutlin-induced apoptosis in tumor cells [122] p53-dependent inhibition of TrxR1 contributes to the tumor-specific induction of apoptosis by RITA [123]. A new therapeutic basis for treating Li-Fraumeni syndrome breast tumors expressing mutated p53 has been suggested [124]. Importang breakthrough was the development of curaxins: anticancer compounds that simultaneously suppress NF-kB and activate p53 by targeting FACT [30, 125, 126].

There was continued development of new ways of drug delivery including liposomes and nanoparticles [91, 127-130]. There are several highly innovative strategies such as targeting tumors with *Salmonella Typhimurium* [131-133] and use of low-level doses of [(32)P]ATP to inhibit tumor growth [134]. Several cancer treatment approaches, such as proteasome inhibitor Bortezomib and hsp90 inhibitor geldanamycin, involve accumulation of misfolded proteins creating proteotoxic stress. Low efficacy of these therapies is likely due to the protective effects of heat shock response (HSR) induced in treated cells, making this pathway an attractive target for pharmacological suppression. It was shown that the anti-malaria drugs quinacrine prevented HSR in cancer cells. Quinacrine did not affect protein synthesis, but rather suppressed inducible HSF1-dependent transcription of the *hsp70* gene. A combination of non-toxic concentrations of quinacrine and proteotoxic stress inducers resulted in rapid induction of apoptosis in cancer cells. Therefore, quinacrine, a non-toxic drug long used for treatment of malaria, has significant clinical potential in cancer therapy [80, 135-137]. Another example is proteotoxic stress targeted therapy (PSTT), where the induction of protein misfolding enhances the antitumor effect of the proteasome inhibitor Bortezomib [138]. Also it was shown that hypoxia enhances the replication of oncolytic herpes simplex virus in p53- breast cancer cells [139].

Aerobic glycolysis, characterized by high glucose uptake, low oxygen consumption and elevated production of lactate, is associated with a survival advantage and is a hallmark of cancer. Targeting key metabolic enzymes involved in glycolysis may provide a novel therapeutic approach [140-145].

There was also further development of the concept of synthetic lethality [146-149]. Synthetic lethal interactions between mutated oncogenes/tumor suppressor genes and molecules involved in DNA damage signaling and repair can be therapeutically exploited to preferentially kill tumor cells [150]. As another example of synthetic lethality, activation of mTOR by targeting TSC2 is toxic in cancer cells lacking Rb [151, 152].

Intriguingly, activation of mTOR converts arrest caused by p53 into senescence [153-156]. And vice versa, inhibition of mTOR allows arrested cells to avoid senescence, remaining merely quiescent. This is in agreement with the notion that mTOR is involved in aging and aging and age-related diseases [157, 158].

There was also further development of the concept of protection of normal cells [159]. Pre-treatment with low doses of actinomycin D, a clinically-approved drug and potent p53 activator, before adding the aurora kinase inhibitor VX-680 protected normal fibroblasts from polyploidy and nuclear morphology abnormalities induced by VX-680 [160]. Similarly, normal cells could be protected from cytotoxic chemotherapy by nutlin-3a, actinomycin, rapamycin and metformin alone or in combinations [161-163]. Several other strategies to protect normal cells are under development [164-166].

As a side effect, CPT-11 can cause severe diarrhea caused by symbiotic bacterial beta-glucuronidases that reactivate the drug in the gut. The strategy was suggested to target these enzymes without killing the bacteria essential for human health. Bacterial beta-glucuronidase inhibitors were identified, which have no effect on the orthologous mammalian enzyme. Inhibitors were effective against the enzyme target in living bacteria, but did not kill the bacteria or harm mammalian cells. Oral administration of an inhibitor protected mice from CPT-11-induced toxicity [167]. In another study, transgenic mice overexpressing p53 were protected from the gastrointestinal syndrome after irradiation. This suggests that the gastrointestinal syndrome is caused by the death of gastrointestinal epithelial cells and that these epithelial cells die by a mechanism that is regulated by p53 but independent of apoptosis [168]. While inhibition of Notch1 plus Notch2 causes severe intestinal toxicity, therapeutic antibody targeting of individual Notch receptors avoids this effect, demonstrating a clear advantage over pan-Notch inhibitors [169]. Interestingly, chromosomal instability (CIN) is associated with intrinsic resistance to taxanes, acquired multidrug resistance and poor prognosis. In contrast, platinum agents may specifically target CIN cancers [170].

In addition to quinacrine, other well known, non-toxic drugs are under re-development for cancer therapy. One of them is metformin, an anti-diabetic drug [171-186]. Several research groups observed that breast cancer patients receiving beta-blockers for hypertension had reduced metastasis and improved clinical outcome. Medical records revealed that beta-blocker treated patients showed a significant reduction in metastasis development, tumor recurrence, and longer disease free interval after surgery. In addition, there was a reduced risk of metastasis and a reduction in breast cancer mortality [187-189]. This finding was further confirmed [190-192]. Another advance is to use of Angiotensin II type 1 receptor blockers in ER-positive and ERBB2-negative breast cancer cases [193].

Thus there have been significant advances to the targeting of various cancers, both with selective inhibitors and with drugs such as rapamycin and metformin which have been used to treat organ transplant patients and diabetics respectively. Further studies will continue to evaluate the effectiveness of targeting the various pathways mentioned in this review with signal transduction inhibitors, natural products, chemotherapeutic drugs and drugs used for different medial purposes either by themselves or in various intelligent combinations based on the knowledge of the critical signal transduction pathways altered in the particular cancer cell.
